# Epigallocatechin-3-gallate targets cancer stem-like cells and enhances 5-fluorouracil chemosensitivity in colorectal cancer

**DOI:** 10.18632/oncotarget.7567

**Published:** 2016-02-22

**Authors:** Shusuke Toden, Hanh-My Tran, Oscar A. Tovar-Camargo, Yoshinaga Okugawa, Ajay Goel

**Affiliations:** ^1^ Center for Gastrointestinal Research, Center for Epigenetics, Cancer Prevention and Cancer Genomics, Baylor Research Institute and Charles A. Sammons Cancer Center, Baylor University Medical Center, Dallas, Texas, USA

**Keywords:** chemoresistance, 5-fluorouracil, colorectal cancer, microRNA

## Abstract

Resistance to cytotoxic chemotherapy is a major cause of mortality in colorectal cancer (CRC) patients. A small subset of cancer cells, termed “cancer stem cells” (CSCs), are believed to be key contributors of chemoresistance and tumor recurrence. Recently, epigallocatechin-3-gallate (EGCG), an active catechin present in green tea, has been shown to suppress CSC growth in various cancers, but whether it can specifically target CSCs and subsequently sensitize chemoresistant CRC cells to standard of care chemotherapeutic treatments remains unknown. Herein, we investigated the chemosensitizing effects of EGCG in 5-fluorouracil (5FU)-resistant (5FUR) CRC cells and spheroid-derived CSCs (SDCSCs), and interrogated the underlying molecular mechanisms responsible for its chemopreventive activity. EGCG enhanced 5FU-induced cytotoxicity and inhibited proliferation in 5FUR cell lines through enhancement of apoptosis and cell cycle arrest. The 5FUR cells showed higher spheroid forming capacity compared to parental cells, indicating higher CSC population. EGCG treatment in these cells resulted in suppression of SDCSC formation and enhanced 5FU sensitivity to SDCSCs. Furthermore, EGCG suppressed Notch1, Bmi1, Suz12, and Ezh2, and upregulated self-renewal suppressive-miRNAs, miR-34a, miR-145, and miR-200c, which are some of the key pathways targeted in 5FUR CRC cells. These findings were validated *in vivo*, wherein EGCG treatment resulted in inhibited tumor growth in a SDCSC xenograft model. Collectively our data provide novel and previously unrecognized evidence for EGCG-induced sensitization to 5FU through targeting of CSCs in CRC. Our data highlight that in addition to its chemopreventive ability, EGCG may serve as an adjunctive treatment to conventional chemotherapeutic drugs in CRC patients.

## INTRODUCTION

Colorectal cancer (CRC) is one of the leading causes of cancer-related deaths in North America [1]. 5-fluorouracil (5FU)-based chemotherapy serves as the first-line, standard of care, chemotherapeutic drug of choice in CRC patients. However, in patients with advanced CRC the response rates to 5FU are merely 0–15% [2], and even combination treatments with oxaliplatin (FOLFOX) or irinotecan (FOLFIRI) yield inadequate response and the majority of the patients fail to respond to these treatments [3, 4]. Furthermore, the majority of chemotherapeutic drug failure in metastatic cancer is attributed to *de novo* or *acquired* chemoresistance [5]. These findings underscore that chemotherapeutic resistance is a major problem in CRC, and the molecular mechanisms underlying this phenomenon remain poorly explored.

Accumulating evidence indicates that a subset of the cancer cell population termed, “cancer stem cells” (CSCs), is a major contributor for resistance to chemotherapeutic agents, and resultant tumor recurrence and metastasis [6]. Classic chemotherapeutic agents are postulated to target differentiated cells, while CSCs appear to escape their toxicity. These data suggest the existence of a significant overlap between signaling pathways involved in drug resistance and self-renewal of cancer cells. In CRC, signaling pathways such as Notch, Wnt, and polycomb repressive complexes (PRC) play a major role in self-renewal regulation [7, 8]. Therapeutic targeting of these pathways to enhance the efficacy of conventional chemotherapy is an attractive strategy in further improvement of treatment response in patients with advanced CRC.

Green tea is a globally popular beverage made from *Camellia sinensis* leaves. In many Asian countries green tea is also used as a traditional medicine to improve blood circulation, wound healing, and digestion. While regular green tea consumption is frequently associated with multiple health benefits, treatment with its principle extract has been shown to reduce formation of metachronous colorectal adenomas [9]. Polyphenols comprise 40% of dried tea leaves, and a major green tea polyphenol, epigallocatechin-3-gallate (EGCG), has been identified as a potent anti-tumorigenic compound [10]. Recently, EGCG has also been shown to inhibit CSCs in breast, glioma, and head and neck cancers [11–13] through suppression of Notch and P-glycoprotein signaling pathways involved in cancer cell self-renewal [12, 13]. However, unlike several other plant-based botanicals, whether EGCG can inhibit formation of CRC CSCs and subsequently contribute to sensitization against chemotherapeutic agents remain unexplored. While conventional therapeutic drugs are somewhat effective at targeting cancer cells, these agents fail to eliminate CSCs. Considering the safety and anti-cancer profile of natural compounds such as EGCG, these polyphenolic agents may provide a safe and cost-effective strategy for targeting CSCs and in reducing chemoresistance and tumor recurrence in CRC patients.

Herein, we firstly demonstrate that EGCG helps overcome chemoresistance to 5FU in chemoresistant CRC cell lines by targeting CSCs. We provide novel evidence that multiple pathways driving self-renewal, including Notch and PRC, were inhibited by EGCG. Furthermore, we identified key tumor suppressive miRNAs that control cancer cell self-renewal to be upregulated following EGCG treatment in 5FU resistant CRC cells. Finally, we used a xenograft animal model to validate our *in vitro* findings and further demonstrate that the combination of EGCG and 5FU significantly reduced tumor proliferation in spheroid-derived CSC tumors. Collectively, these data indicate that in addition to its cancer preventive properties, EGCG may serve as an adjunct to conventional chemotherapy in colorectal cancer.

## RESULTS

### EGCG enhances sensitivity to 5FU in 5FUR colorectal cancer cells

**Figure 1:**
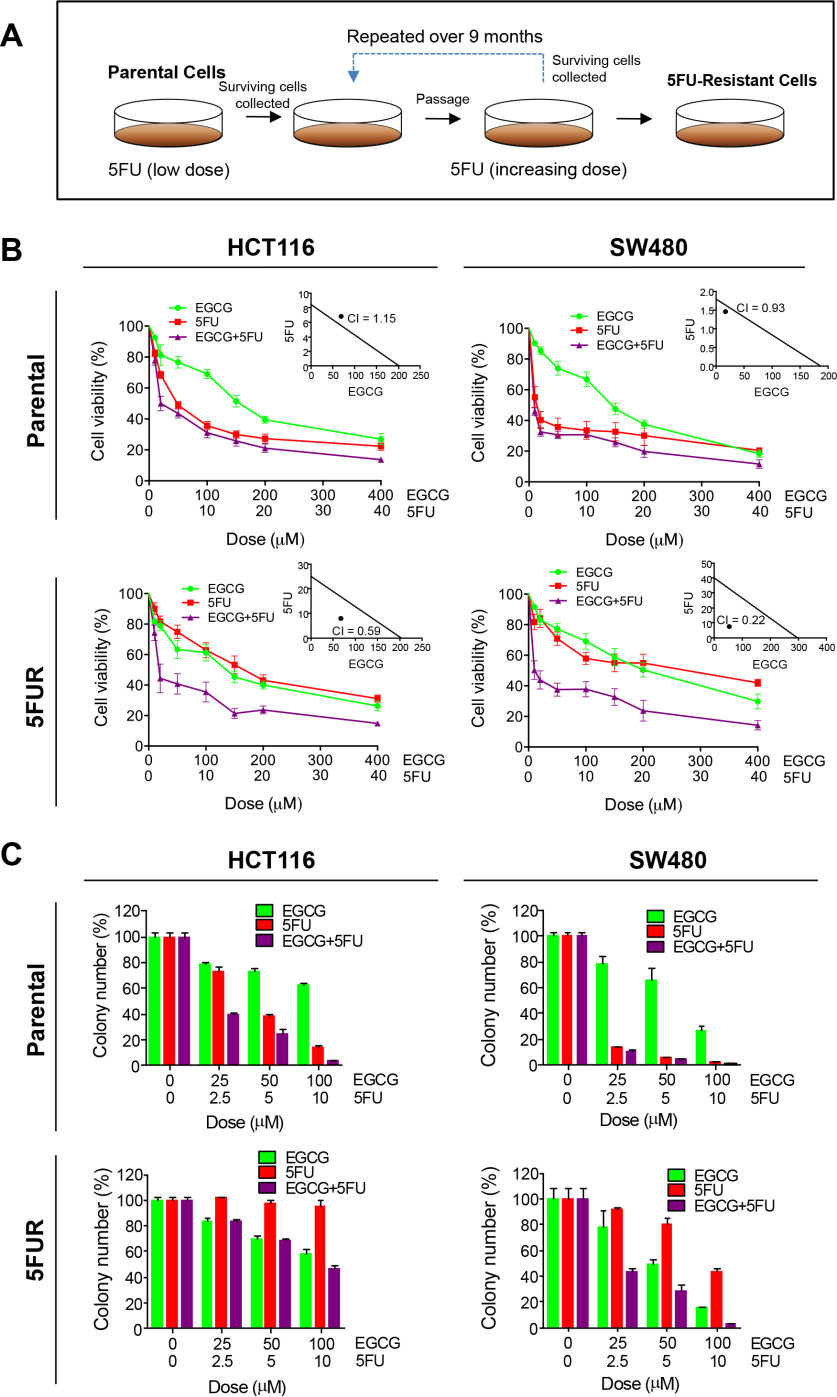
EGCG enhances 5FU sensitivity in 5FU resistant colorectal cancer cells (**A**) Schematic diagram for the establishment of chemoresistant cell lines. (**B**) Cytotoxicity of EGCG and 5FU in HCT116 and SW480 and their respective resistant counterpart cell lines treated with 25–400 μM of EGCG and/or 2.5–40 μM 5FU. Insert: Synergy between EGCG and 5FU was calculated by combined index (CI). (**C**) Colony formation assays of HCT116 and SW480 and their respective 5FUR cells treated with EGCG and/or 5FU. *P < 0.05, **P < 0.01 ***P < 0.001 compared to control.

In order to determine whether EGCG enhances the efficacy of 5FU, we measured the cytotoxicity of both compounds individually and in combination using both parental and 5FUR HCT116 and SW480 cell lines. We first determined appropriate experimental doses for both EGCG and 5FU in CRC cell lines. 5FU was approximately 10 times more potent than EGCG in the resistant cell lines, hence we used a 1:10 ratio for the combined treatment. 5FU caused greater cytotoxicity than EGCG in both parental cell lines, while the combination of the two compounds showed minor enhancement in cytotoxicity. Chou-Talalay combination index revealed that the combined EGCG and 5FU treatment resulted in weak or no synergistic effects, indicating that EGCG does not enhance the chemotherapeutic potential of 5FU in parental cell lines (Figure 1B insert). To determine the effects of EGCG and 5FU on CRC cell lines with 5FU resistance, we generated 5FU resistance (5FUR) cells by treating these cells with increasing concentrations of 5FU over duration of 9 months. Following treatment these cells acquired mesenchymal like appearance and enhanced expression of oncogenes including ZEB1 and BMI1 [14] and enhanced resistance to apoptosis through alteration of apoptosis related genes [15]. In the 5FUR cell lines the combined treatment resulted in significant synergistic enhancement in cytotoxicity (Figure 1B insert). Collectively these data suggest that ECGC could attenuate 5FU resistance in 5FUR cell lines. Next, we evaluated the combinatorial effects of EGCG and 5FU on cell growth and survival using colony formation assays. In the parental HCT116 and SW480 cells, both 5FU and EGCG inhibited colony formation, while the combined treatments effectively suppressed colony formation at a significantly lower dose (Figure 1C). While 5FU treatment was less effective in reducing colony formation capacity in 5FUR cell lines, the addition of EGCG significantly enhanced the ability of 5FU to inhibit colony formation (Figure 1C) in both CRC cell lines.

### EGCG induces apoptosis and cell cycle arrest in 5FU-resistant colorectal cancer cells

**Figure 2:**
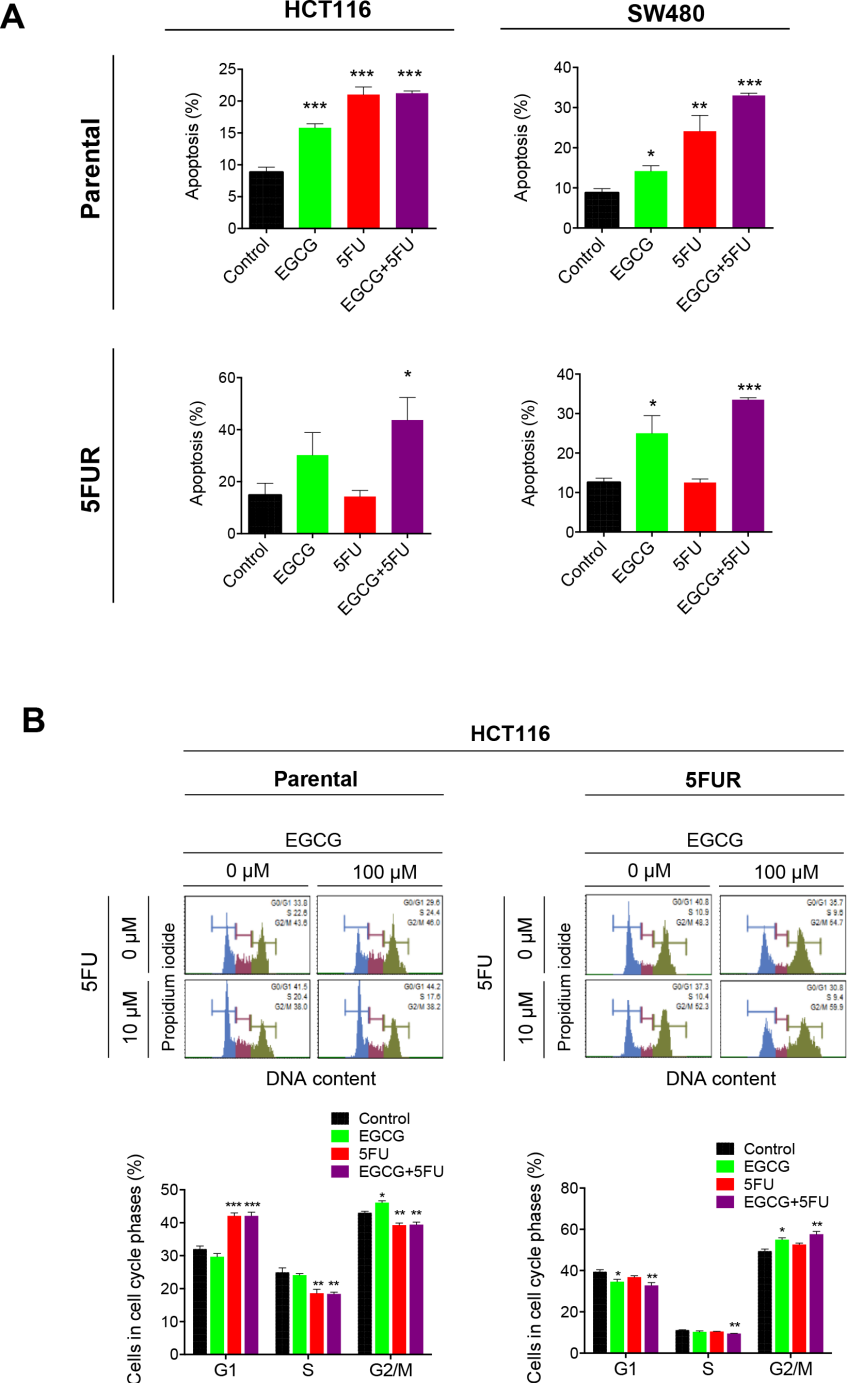
EGCG induces apoptosis and cell cycle arrest in 5FU resistant colorectal cancer cells (**A**) Cells were stained with Annexin V and 7-AAD, and apoptotic cell number was determined by flow cytometry. (**B**) Cell cycle analysis for cells treated with EGCG and/or 5FU, followed by staining with propidium iodide and subjected to flow cytometry analysis for the determination of DNA content. *P < 0.05, **P < 0.01 ***P < 0.001 compared to control.

Next we examined whether the synergistic interaction between EGCG and 5FU in cellular growth inhibition resulted in corresponding increase in programmed cell death. Both EGCG and 5FU treatment significantly enhanced cellular apoptosis in parental HCT116 and SW480 cells, while combined EGCG and 5FU treatment further increased apoptosis in parental cell lines (Figure 2A). The enhanced apoptosis in the combined treatment appeared to be primarily driven by 5FU in the parental cell lines. In contrast, treatment with 5FU did not induce apoptosis in both 5FUR cell lines, but the addition of EGCG in 5FUR cells lines caused significant enhancement in cellular apoptosis. These data confirm establishment of acquired resistance to 5FU in 5FUR cell lines (Figure 2A).

We then investigated how ECGC and 5FU regulate cell cycle in both parental and 5FUR CRC cell lines. 5FU is a potent inducer of cell cycle arrest capable of inducing both G0/G1 and G2 arrest in CRC cells [16]. In the HCT116 parental cell line, 5FU treatment induced G0/G1 arrest, while EGCG induced G2 arrest (Figure 2B). The combined treatment with EGCG and 5FU resulted in G0/G1 arrest, suggesting that 5FU is primarily responsible for the cell cycle regulation in the parental cells. In contrast, 5FU treatment had no effect on cell cycle dynamics in 5FUR cells, while EGCG treatment resulted in a G2 growth arrest. The combined EGCG and 5FU treatment resulted in enhanced G2 phase arrest indicating that EGCG is the primary driver of cell cycle arrest in 5FUR cells.

### EGCG inhibits cancer stem cell formation in colorectal cancer cells

**Figure 3:**
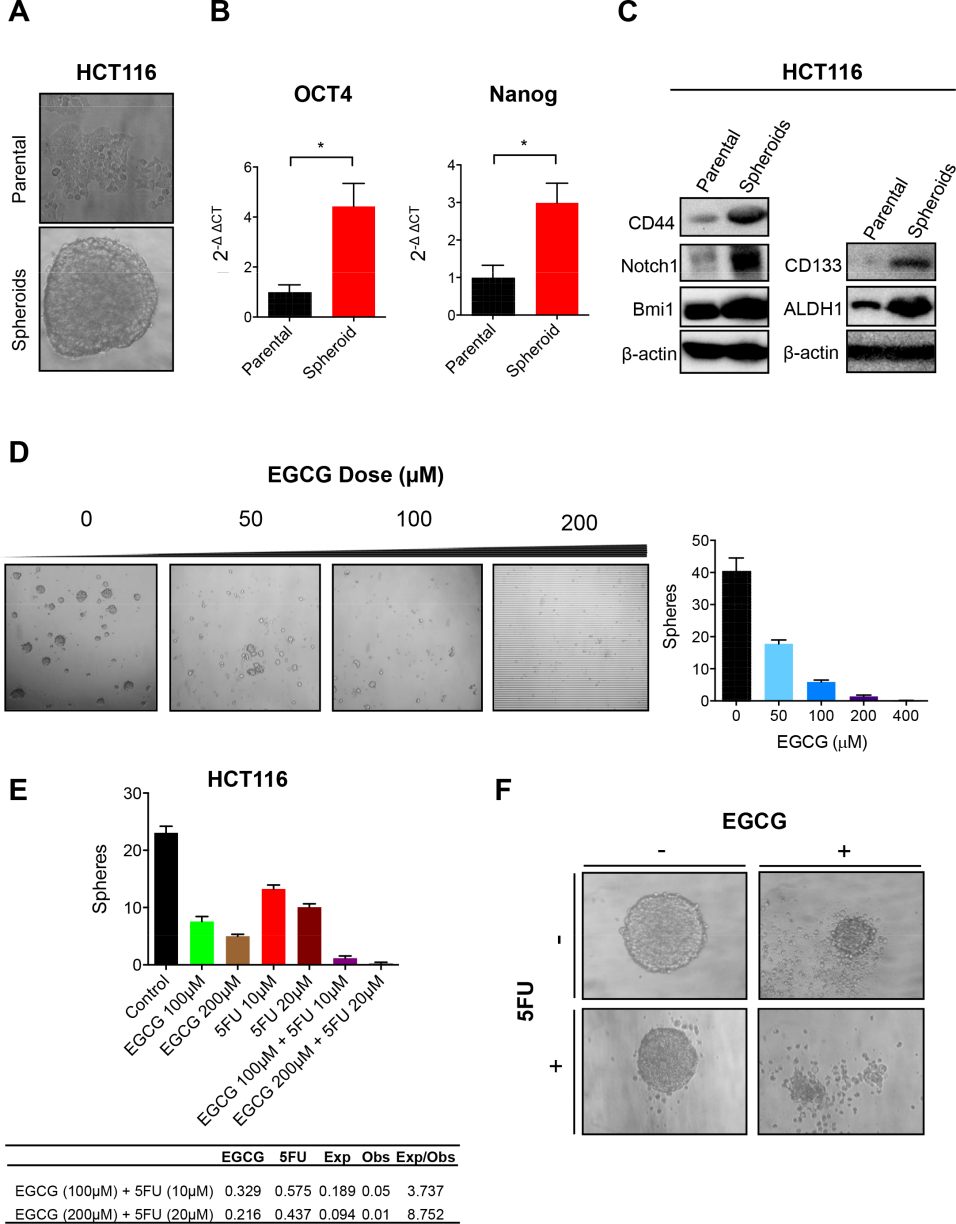
EGCG attenuates spheroid derived cancer stem cell formation in colorectal cancer cells (**A**) Image of HCT116 parental cells and spheroid-derived CSC. (**B**) Oct4 and Nanog expression in HCT116 parental and SDCSC, normalized to GAPDH. (**C**) Western blot analysis of CD44, Notch1, and Bmi1 treated with EGCG and/or 5FU in HCT116 parental or SDCSC. (**D**) SDCSC treated with various doses of EGCG (0–100) representative image left, quantified data right. (**E**) SDCSC treated with EGCG and/or 5FU. Fraction of sphere number (FSN) was calculated for both EGCG and 5FU treatment alone in the table. Expected FSN (Exp FSN) was calculated by multiplying the FSN of EGCG and 5FU then comparing it to the observed FSN (Obs FSN). Ratio of Exp FSN/Obs FSN > 1 indicates a synergistic effect. (**F**) Images of spheroids treated with EGCG and/or 5FU

Considering that CSCs have been postulated as the major contributors of chemoresistance, we investigated whether EGCG can attenuate CRC spheroid formation. We used HCT116 parental cells to generate spheroids (Figure 3A). These SDCSCs showed significantly higher expression of stem cell markers, Oct4 and Nanog, compared to the parental cells (both *P* < 0.05) (Figure 3B). We then assessed protein expression of CD44, a well-established surface stem cell marker, and self-renewal markers Notch1 and Bmi1. As expected, CD44, Notch1, Bmi1, CD133 and ALDH1 expression was upregulated in SDCSCs compared to parental cells, indicating that these spheroids have a higher CSC population (Figure 3C). Next, we investigated whether HCT116 cells can form spheroids in a medium containing various doses of EGCG. 50 μM EGCG treatment reduced spheroid formation by over 50% and further reduced the spheroid numbers with increasing concentrations of EGCG (Figure 3D). We then treated pre-grown spheroids with EGCG and/or 5FU to determine whether these agents can synergistically inhibit sphere forming capacity. While individual treatment with EGCG or 5FU suppressed the number of spheroids, the combined treatment further inhibited sphere forming capacity. In order to determine whether this reduction by the combined treatment was synergistic, we calculated the expected sphere numbers using fraction of sphere number (FSN) in both EGCG and 5FU treated cell lines (Figure 3E). Interestingly, the expected:observed FSN ratio for 100 μM EGCG + 10 μM 5FU and 200 μM EGCG + 20 μM 5FU was 3.737 and 8.752 respectively, indicating a synergistic reduction in sphere number. Microscopic analysis showed that more spheroids underwent apoptosis when treated with both EGCG and 5FU (Figure 3F), indicating the affinity of EGCG in inhibiting SDCSC formation in 5FUR cells.

### EGCG inhibits Notch pathway and polycomb repressive complex subunits in 5FU resistant colorectal cancer cells

**Figure 4:**
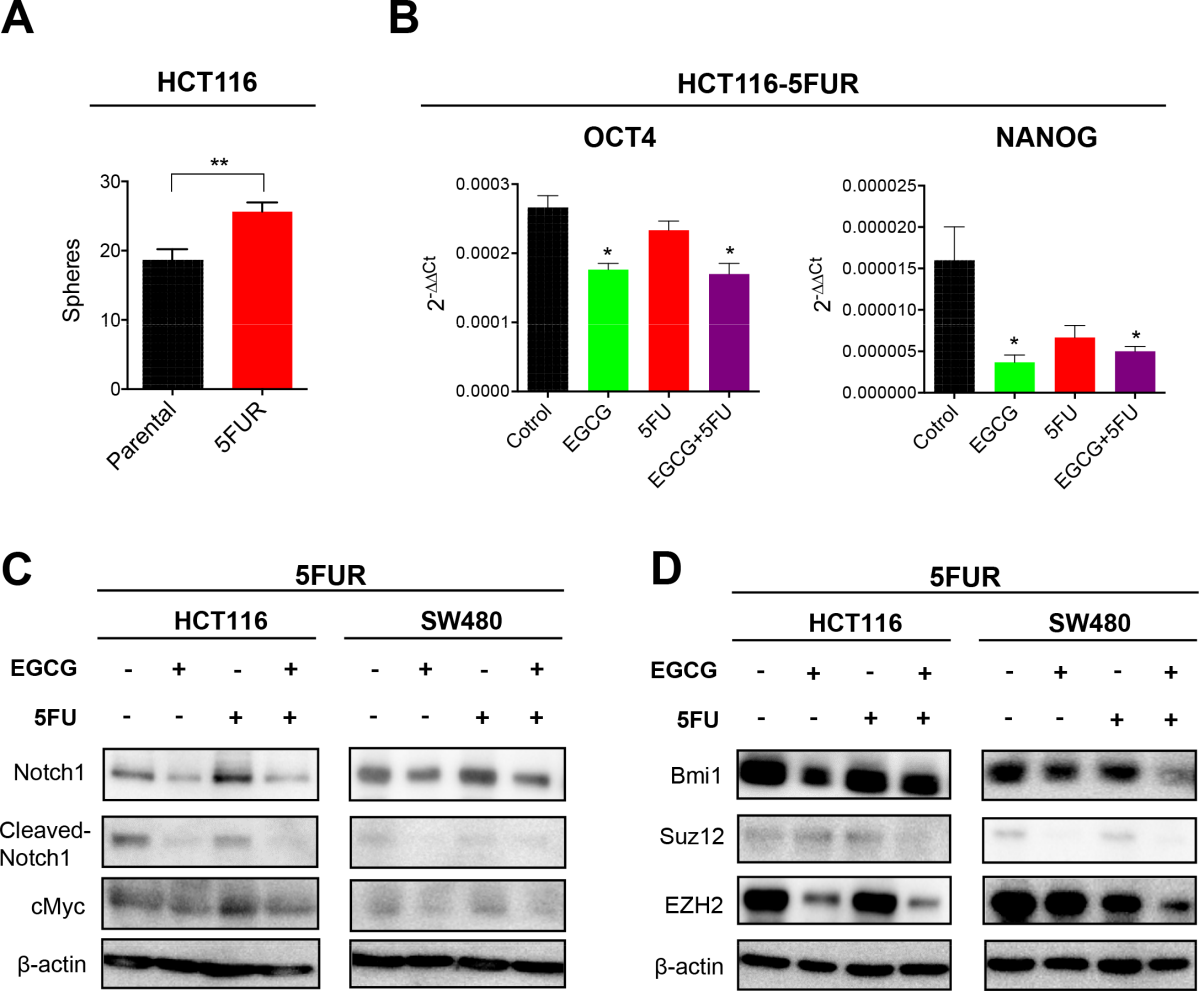
EGCG inhibits Notch signaling pathways and polycomb repressive complex subunits in colorectal cancer cells (**A**) Sphere forming capacity of HCT116 parental and 5FUR cells. (**B**) Oct4 and Nanog expression in 5FUR cell lines treated with EGCG and/or 5FU. (**C**) Protein immunoblot analysis of Notch1, cleaved-Notch 1, and cMyc in 5FUR cell lines treated with EGCG and/or 5FU. (**D**) Protein immunoblot analysis of Bmi1, Suz12, and Ezh2 in 5FUR cell lines treated with EGCG and/or 5FU.

Considering 5FUR cells have higher resistance to 5FU, we assessed the sphere forming capacity of 5FUR cells compared to their parental counterparts. Consistent with a previous study using drug resistant CRC cells [17], 5FUR CRC cells demonstrated a higher sphere forming capacity compared to parental cells (Figure 4A). The expression of stem cell markers, Oct4 and Nanog, were both downregulated by EGCG treatment, indicating that EGCG suppressed CSC formation in 5FUR cells (Figure 4B). The Notch pathway is a key regulatory signaling pathway for self-renewal, and has been shown to expand the population of proliferating intestinal progenitor cells by inhibiting cell differentiation [18]. Since EGCG has been shown to suppress the Notch pathway in head and neck cancer [12], we examined whether EGCG suppresses the Notch signaling pathway individually or in combination with 5FU in 5FUR CRC. While Notch1 expression was downregulated by both EGCG and the combined treatments in both HCT116 and SW480 5FUR cell lines, 5FU treatment by itself did not alter Notch1 expression (Figure 4C, Supplementary Figure 1). Likewise, the expression of cleaved-Notch1, the active component of Notch1 that triggers nuclear translocation, was downregulated by EGCG, but not by 5FU treatment (Figure 4C, Supplementary Figure 1). We next assessed the effects of EGCG on cMyc, a well-established oncogene that is also one of pluripotency maintenance transcription factors [19, 20]. The expression of cMyc was downregulated by EGCG treatment alone and addition of 5FU further downregulated the expression of cMyc (Figure 4C, Supplementary Figure 1). Next we investigated alterations in the expression of various key PRC subunits linked to generation of CSCs. Dysregulation of polycomb proteins results in activation of developmental pathways, thereby enhancing proliferation capacity and drives CSC formation [21]. In particular, Bmi1 has been suggested to have a significant role in chemoresistance and tumor recurrence [22]. We noted that the expression of Bmi1, Ezh2, and Suz12 were downregulated by EGCG in 5FUR cell lines, while 5FU treatment alone did not have any impact on the expression of these genes (Figure 4D, Supplementary Figure 1). Collectively these data suggest that EGCG may attenuate chemoresistance of cancer cells through interception of multiple key signaling pathways involved in self-renewal.

### EGCG upregulates the expression of key tumor suppressive microRNAs in colorectal cancer cells

**Figure 5:**
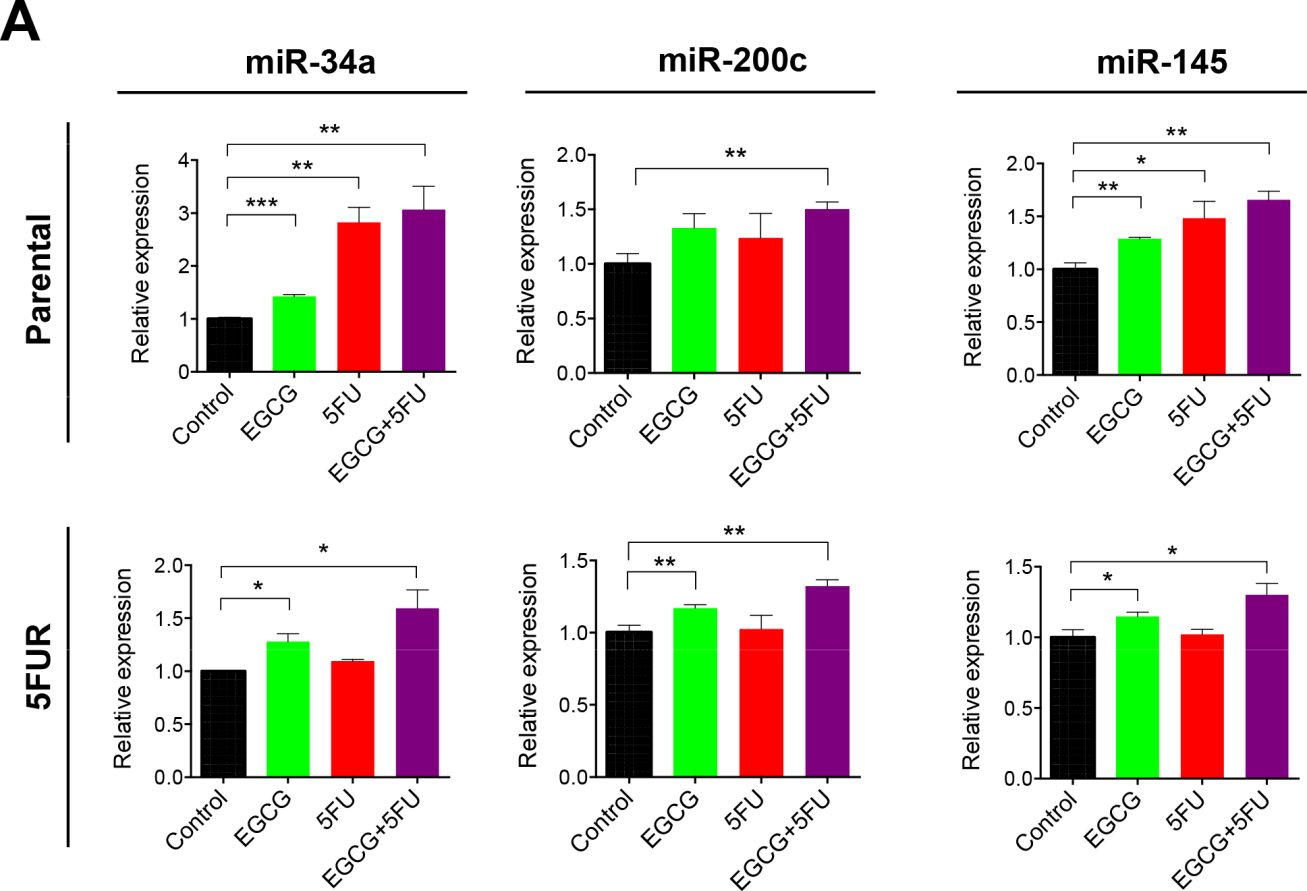
EGCG upregulates key self-renewal/tumor suppressive miRNAs in colorectal cancer cells The expression of miR-34a, miR-145 and miR-200c were assessed in HCT116 parental and 5FUR cell lines treated with EGCG and/or 5FU and normalized to RNU6B. *P < 0.05, **P < 0.01 ***P < 0.001 compared to control.

Next we investigated whether EGCG can modulate the expression of key tumor suppressive miRNAs known for their regulation of self-renewal capacity. MiR-34a, miR-145, and miR-200c are three well-established tumor suppressive miRNAs known for inhibiting self-renewal [23–25]. MiR-34a, in particular, has been known to act as the suppressor of symmetric colon CSCs and asymmetric division [25]. In our study, miR-34a expression was upregulated primarily by 5FU treatment in parental cells, but this treatment had no effect on miR-34a expression in 5FUR cells (Figure 5). In contrast, EGCG treatment resulted in the upregulation of miR-34a in both parental and 5FUR cell lines. Similarly, loss of miR-200c expression has been linked with cancer progression and chemoresistance through CSC generation and induction of epithelial-to-mesenchymal transition. Furthermore, miR-200c functions as a tumor suppressor by directly inhibiting PRC oncogenes, Bmi1 and Suz12 [26, 27]. EGCG treatment resulted in significant upregulation of miR-200c expression in both 5FUR cell lines (Figure 5). In addition, another tumor suppressive miRNA, miR-145 was upregulated by EGCG in both parental and resistant cell lines, while 5FU treatment by itself did not alter miR-145 expression in both cell lines. These data indicate that EGCG treatment regulated expression of key miRNAs that control cancer cell self-renewal through suppression of common target genes in 5FU resistant colorectal cancer cells.

### Combined EGCG and 5FU treatment attenuates tumor formation in SDCSC xenografts

**Figure 6:**
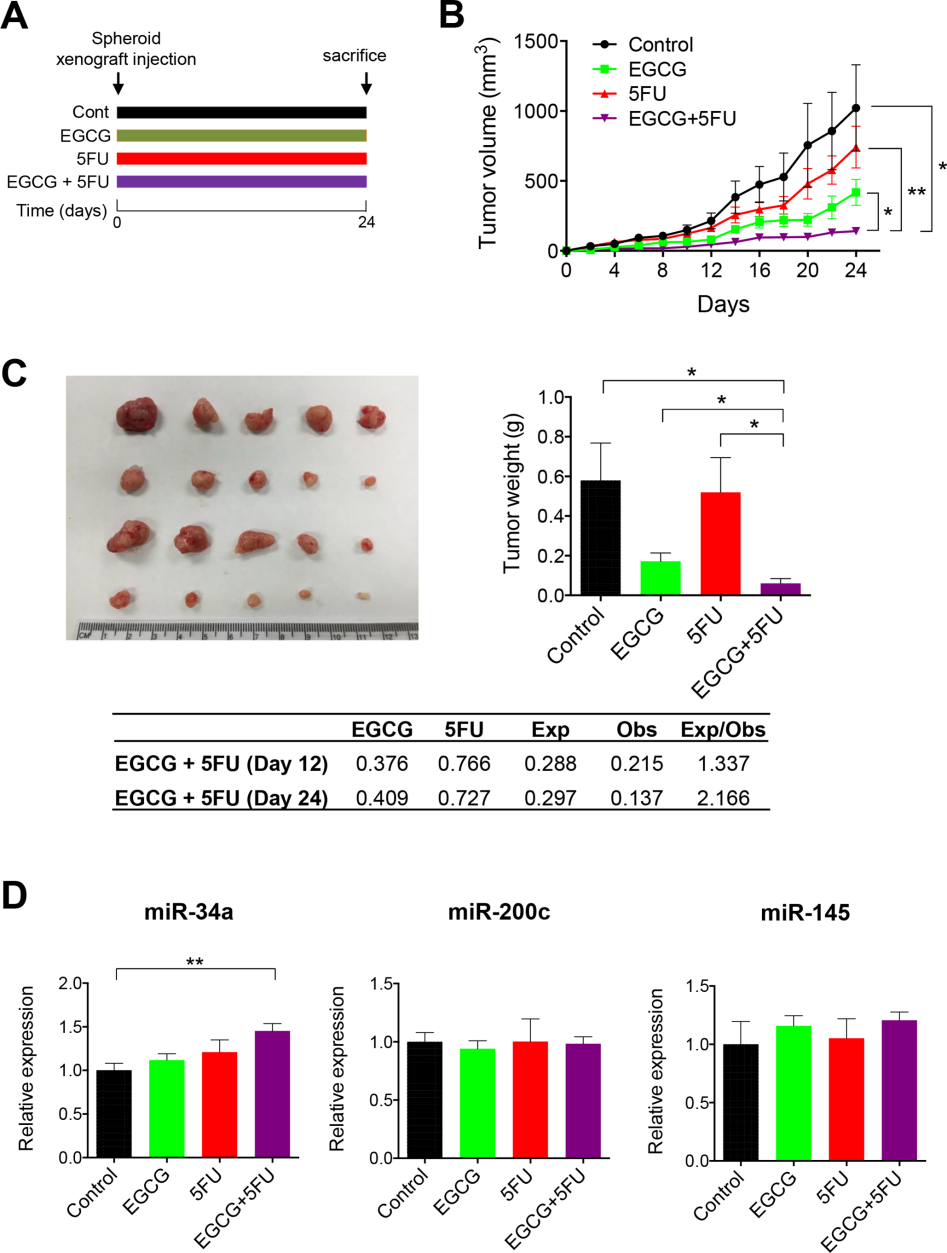
EGCG enhances 5FU against 5FUR derived xenograft tumors (**A**) The schematic diagram of the EGCG and 5FU treatment protocol. (**B**) Progressive xenograft tumor volume. (**C**) Xenograft tumors collected from experimental animals (left) and average tumor weight with treatments (right)/Fraction of tumor volume (FTV) was calculated for both EGCG and 5FU treatment alone in the table. Expected FTV (Exp FTV) was calculated by multiplying the FTV of EGCG and 5FU, then comparing it to the observed FTV (Obs FTV). Ratio of Exp FTV/Obs FTV > 1 indicates a synergistic effect. (**D**) The expression of miR-34a and miR-200c were assessed in xenograft tumors. *P < 0.05, **P < 0.01 ***P < 0.001.

Finally, we determined whether EGCG and 5FU can attenuate tumor growth individually or in combination in a SDCSC xenograft model (Figure 6A). EGCG suppressed SDCSC generated tumor growth and resulted in lower tumor weight compared to DMSO controls (Figure 6B and 6C). Furthermore, the combined EGCG and 5FU treatment had superior tumor growth inhibition compared to EGCG treatment alone. Next, we determined whether the combination treatment resulted in synergistic reduction in tumor volume (ratio of expected:observed FTV > 1). FTV values at both day 12 and 24 were 1.337 and 2.166 respectively, indicating the synergistic reduction of xenograft tumor growth. In addition, we examined miRNA levels from xenograft tumors. While miR-34a was significantly upregulated in the combined treatment (*P* < 0.01), miR-200c expression was not significantly different between the treatment groups (Figure 6D). Taken together, these data confirmed our *in vitro* findings for EGCG-induced enhancement of 5FU cytotoxicity in colorectal cancer.

## DISCUSSION

In this study, we for the first time demonstrate a novel molecular mechanism for EGCG-induced chemosensitization to 5FU in CRC by targeting the cancer stem cell population. Herein we showed that 5FUR cells have a higher spheroid forming capacity compared to parental cells, indicating that 5FUR cells have greater self-renewal capacity. However, when treated with EGCG, this polyphenol suppressed SDCSC formation, suggesting its ability to block CSC formation in CRC. Furthermore, EGCG treatment downregulated key cell signaling pathways involved in self-renewal including Notch and PRC, which is controlled by the upregulation of expression of specific miRNAs that target these genes. In addition, we confirmed our *in-vitro* findings using a CSC-derived xenograft model, highlighting the potential therapeutic usefulness of EGCG as an adjunctive treatment to 5FU in colorectal cancer patients.

The present study builds upon the premise that EGCG treatment has the potential to target CSCs, leading to enhanced cytotoxicity in 5FU resistant CRC cells. While CSCs may be perceived as a small fraction of the total cancer cell population, these cells display high sphere forming capacity and are postulated as drivers for acquired chemoresistance in cancer patients [28]. Since most conventional chemotherapeutic drugs interfere with the growth of rapidly dividing cancer cells, it is believed that CSCs are spared from these treatments, leading to chemoresistance, metastasis, and tumor recurrence. The Notch signaling pathway is frequently overexpressed in colon CSCs and is believed to be responsible for colon CSC formation and self-renewal [29, 30]. This pathway regulates cancer cell self-renewal and differentiation, and interception of Notch signaling has already been proposed to be a potentially attractive therapeutic strategy in CRC [31]. In this study, we demonstrated that EGCG suppresses Notch1 and cleaved Notch1 in 5FUR cell lines indicating that EGCG treatment can inhibit the Notch signaling pathway in CRC - a finding that is consistent with a previous study conducted in head and neck cancer [12]. Moreover, we also demonstrated that EGCG downregulates key PRC subunits including Bmi1, Ezh2, and Suz12 in 5FUR cell lines. The polycomb group is a class of chromatin modifying enzymes that regulate gene expression by methylating both DNA and core histones, and this process has been shown to directly regulate developmental factors that maintain embryonic stem cell, self-renewal, and pluripotency [32]. PRC subunits such as Bmi1 and Ezh2 are often over-expressed in various cancers [33] and are required for formation and maintenance of CSCs [21]. In particular, Bmi1 has been identified as a key therapeutic target in CRC [34, 35], and has been shown to be overexpressed in drug resistant breast cancer [36]. Our present study demonstrated that EGCG downregulates Bmi1 in 5FUR cells, and this could be a critical molecular mechanism by which EGCG suppresses self-renewal capacity and subsequently sensitizes CRC cells to 5FU-based chemotherapeutic treatment. In addition, our data for the first time revealed that transcription factors, *NANOG*, *OCT4* and *MYC*, which are required for maintenance of pluripotency in stem cells [37], were significantly suppressed by EGCG treatment in 5FUR cells. These findings are consistent with a previous study that assessed pluripotency transcription factors in pancreatic CSCs [38]. Collectively, we demonstrated that EGCG inhibits multiple self-renewal pathways in 5FUR CRC, resulting in enhanced sensitivity to 5FU-based chemotherapy.

Even though many current therapeutic strategies aim to target a specific self-renewal pathway using a unique drug/inhibitor, such compounds are unlikely to be effective considering the involvement of multiple interactive pathways that drive self-renewal capacity in CRC [7]. Hence targeting one specific pathway will allow cancer cells to escape via alternative pathways, and such approaches will be inadequate to eliminate CSCs. Our data demonstrated that EGCG suppresses several major self-renewal driving pathways in CRC. When these results are considered together with other reports in breast and pancreatic cancers, where EGCG has been shown to also inhibit Wnt and sonic hedgehog pathways [38, 39], it is obvious that EGCG is a potent botanical that can selectively block self-renewal pathways, and has promise and potential clinical application.

One of the main causes of death from CRC is liver metastasis. Emerging evidence indicates that probability of liver metastasis is highly dependent on CSCs [40, 41]. The present study, along with other previous reports, has demonstrated that EGCG targets CSCs in various cancers [11–13]. The ability of EGCG to target CSCs has significant clinical implication as cytotoxic chemotherapeutic agents are not effective in targeting CSCs. Hence, compounds derived from natural botanicals such as EGCG could be used therapeutically in conjunction with conventional chemotherapy drugs for patients with high risk of metastasis.

MiRNAs are short non-coding RNAs that regulate expression of multiple genes through biding to the 3′-untranslated region of various genes. These non-coding RNAs play a significant role in oncogenesis including regulation of self-renewal and embryonic development [42, 43]. Tumor suppressive-miRNA, miR-34a, is downregulated in drug-resistant pancreatic cancer cell lines, and ectopic overexpression of miR-34a has been shown to induce chemo-sensitization to camptothecin [44]. Similarly miR-200c expression has been linked to cancer progression and chemoresistance via modulation of epithelial-to-mesenchymal transition [27, 45]. A recent study identified an inhibitory feedback loop between miR-200c and Bmi1 that could have a major role in regulation of chemoresistance [36]. Our findings demonstrated that both tumor suppressive miRNAs, miR-34a and miR-200c, were upregulated by EGCG which corresponded with downregulation of their target genes *MYC*, *BMI1*, and *SUZ12*. In addition, we showed that the expression of tumor suppressive miR-145 was upregulated by EGCG in 5FUR cells. MiR-145 is another miRNA known for inhibition of self-renewal through suppression of Oct4 and Adam17, an enzyme involved in the Notch pathway [24, 46]. While the increase in the expression of these tumor suppressive miRNAs was relatively modest, nonetheless, EGCG was able to regulate the expression of multiple miRNAs and their downstream target genes. Considering that a single miRNA is capable of modulating hundreds of genes, including unwanted genes, therapeutic use of miRNAs requires careful evaluation. Alteration of miRNA expression by botanical treatment is generally small, but provides a guideline for naturally occurring miRNA alteration. Interestingly, since miRNAs interact with multiple target genes, coordinated regulation of multiple miRNAs could minimize unwanted upregulation of oncogenes – an approach that was recently demonstrated in pancreatic cancer [47]. These findings highlight that targeting of multiple miRNAs for CRC treatment may have a significant advantage over a use of specific miRNA inhibitor or a mimic.

In summary, we demonstrated for the first time that EGCG enhances 5FU sensitivity in chemoresistant CRC by targeting CSCs. Herein, we provide novel insights into the molecular mechanisms underpinning the biology behind chemoresistance in CRC, and demonstrate that EGCG inhibits multiple self-renewal driving pathways including Notch and Bmi1, Ezh2, and Suz12, through upregulation of the expression of key tumor suppressive miRNAs. In view of the limitations of the current generation of chemotherapeutic drugs that are inefficient at targeting CSCs, the use of natural products like EGCG in CRC may provide a safe and effective adjunctive approach in overcoming therapeutic resistance in CRC.

## MATERIALS AND METHODS

### Cell culture and materials

Both EGCG and 5FU were purchased from Sigma-Aldrich (Sigma-Aldrich, St. Louis, MO) and dissolved in DMSO and diluted to appropriate experimental concentrations with tissue culture medium. HCT116 and SW480 CRC cells were purchased from American Type Culture Collection (Manassas, VA), and 5FU resistant (5FUR) cell lines were established by a previously described method [48] by treating these cells with increasing concentrations of 5FU over a duration of 9 months (Figure 1A). All cell lines were routinely interrogated for candidate genetic and epigenetic biomarkers to confirm their authenticity. The cells were grown in Dulbecco's Modified Eagle Medium (DMEM; Invitrogen, Carlsbad, CA), supplemented with 10% fetal bovine serum, 1% penicillin and streptomycin, and maintained at 37°C in a humidified incubator at 5% CO_2_. 5FUR cells were maintained in culture medium containing 5 μM 5FU. CRC spheroid-derived cancer stem cells (SDCDC) were generated from HCT116 cells in serum free medium (DMEM/F12) supplemented with B27, N2 supplements (Gibco, Invitrogen, Carlsbad, CA), 10 ng/ml human recombinant basic fibroblast growth factor (bFGF, Gibco), and 20 ng/ml epidermal growth factor (EGF, Sigma-Aldrich) and cultured in Coster^®^ ultra-low attachment flask (Corning, Corning, NY).

### Viability, cell cycle, apoptosis and colony formation assays

Cellular cytotoxicity was determined using the 3-(4,5-dimethylthiazole-2-yl_2,5-diphenyl tetrazolium bromide (MTT)) assay as described previously [49]. Cells were incubated with various concentrations of EGCG and/or 5FU for 72 hours. Optical density was determined using the Infinite 200 Pro multi-reader and i-control 1.10 (Tecan Group Ltd, Mannedorf, Switzerland). The combination index (CI) was calculated using the Chou-Talalay equation [50] at 50% inhibitory concentration to determine synergism between EGCG and 5FU. Cell cycle analysis was conducted using the Cell Cycle Assay Kit (MCH100106; Millipore, Billerica, MA), and apoptotic cell fraction was measured using the Annexin V and Dead Cell Assay Kit (MCH100105; Millipore) on Muse Cell Analyzer (Millipore) according to the manufacturer's instructions. In addition, colony formation assays were conducted as described previously [51]. The number of colonies (> 50 cells) were counted using GeneTools (Syngene, Cambridge, UK). All experiments were conducted in replicates and at least three independent times.

### Quantitative real-time PCR analysis

Expression of miRNAs was analyzed using the TaqMan^®^ real-time PCR assay kit (Applied Biosystems, Foster City, CA) as described previously [52]. Cells were treated with EGCG (100 μM) and/or 5FU (10 μM) for 24 hours, and RNA was extracted using the miRNeasy Mini Kit (Qiagen, Germantown, MD). For all reactions, TaqMan^®^ Universal Master Mix was used and the analysis was carried out using StepOnePlus system (Applied Biosystems). All data were analyzed using ΔΔCt method and normalized to RNU6B. For analysis of the mRNA expression, 1 μg of total RNA was reverse transcribed to complimentary DNA using Advantage RT PCR-kit (Clonotech Laboratories Inc., Mountain View, CA). Power SYBR Green (Applied Biosystems) real-time PCR was performed using StepOnePlus system. For specific primer sequences refer to Supplementary Table 1. The expression of qRT-PCR amplified target genes were normalized to glyceraldehyde-3-phosphate dehydrogenase (GAPDH) and ACTB using previously described method [53], and results were analyzed using the ΔΔCt method.

### Western blot

Western immunoblotting experiments were performed as described previously [54]. Cells were treated with ECGC and/or 5FU for 24 hours and lysed using 100 μl of 1 X SDS sample buffer containing β-mercaptoethanol. The list of primary antibodies is provided in Supplementary Table 2, and anti-mouse or anti-rabbit antibodies (Santa Cruz Biotechnology, Santa Cruz, CA) were used as secondary antibodies. All samples were compared against β-actin (Sigma-Aldrich) as a reference protein. The protein bands on the gels were visualized (G:Box, Syngene) and protein density was measured using GeneTools (Syngene).

### Sphere forming assay

Cells were dissociated into single cells, and seeded in a Coster^®^ ultra-low attachment 96-well plate (Corning) in serum free stem cell medium. Spheroids were treated with EGCG and/or 5FU 24 hours after initial seeding. Spheres were counted using a light microscope (Olympus, Tokyo Japan) after 7 day incubation. The interaction between EGCG and 5FU on sphere forming capacity was evaluated as described previously [55]. The fraction of sphere number (FSN) affected by EGCG and/or 5FU was calculated individually and in combination, and represented as ratios to control. The ratio of expected FSN (Exp FSN) and observed FSN (Obs FSN) was calculated for the combined treatment (A ratio > 1 indicates synergistic effect, < 1 indicates an additive effect).

### Animal experiments

The 5 week-old male athymic nude mice (Harlan Laboratories, Houston, TX) were housed under controlled conditions of light and fed *ad libitum*. Xenograft tumors were generated by injecting HCT116-SDCSCs treated for 48 hours with DMSO, EGCG (100 μM), 5FU (10 μM), and the combination of both EGCG and 5FU. Thereafter, 2 × 10^6^ HCT116-SDCSCs suspended in Matrigel matrix (BD Biosciences, Franklin Lake, NJ) were subcutaneously injected into flanks of mice using a 27-gauge needle. Tumor size was measured every other day by calipers for 24 days. Tumor volume was calculated using the following formula: 1/2 (length × width × width). The interaction between EGCG and 5FU on tumor volume was evaluated as described previously [55]. The fraction of tumor volume (FTV) affected by EGCG and/or 5FU was calculated individually and for the combination treatment by determining ratios between treatment vs. controls. The ratio of expected FTV (Exp FTV) and observed FTV (Obs FTV) was calculated for the combined treatment (A ratio > 1 indicates synergistic effect, < 1 indicates an additive effect). All tumor samples were dissected and stored in RNAlater (Sigma-Aldrich) for subsequent analysis. The animal protocol was approved by the Institutional Animal Care and Use Committee, Baylor Research Institute, Dallas, Texas.

### Statistical analysis

All analyses were performed using GraphPad Prism Ver. 6.0 (GraphPad Software Inc. San Diego, CA). All data were expressed as mean ± SEM with statistical significance indicated when *P* < 0.05. Statistical comparisons between control and treatment groups were determined using unpaired *t* test or one-way ANOVA with Tukey's post-hoc tests

## SUPPLEMENTARY MATERIALS FIGURE AND TABLES



## Abbreviations

EGCG: epigallocatechin-3-gallate
5FU: 5-fluorouracil
5FUR: 5-fluorouracil resistance
CRC: colorectal cancer
CSC: cancer stem cells
SDCSC: spheroid-derived cancer stem cells
