# Supplementary material for: Epigallocatechin-3-gallate targets cancer stem-like cells and enhances 5-fluorouracil chemosensitivity in colorectal cancer
Source: Oncotarget. 2016 Feb 22;7(13):16158–71. doi: 10.18632/oncotarget.7567 (PMC4941304; doi:10.18632/oncotarget.7567)
Supplement: Supplementary file 1 [file oncotarget-07-16158-s001.pdf]

# Epigallocatechin-3-gallate targets cancer stem-like cells to enhance 5-fluorouracil chemosensitivity in colorectal cancer

## Supplementary Materials

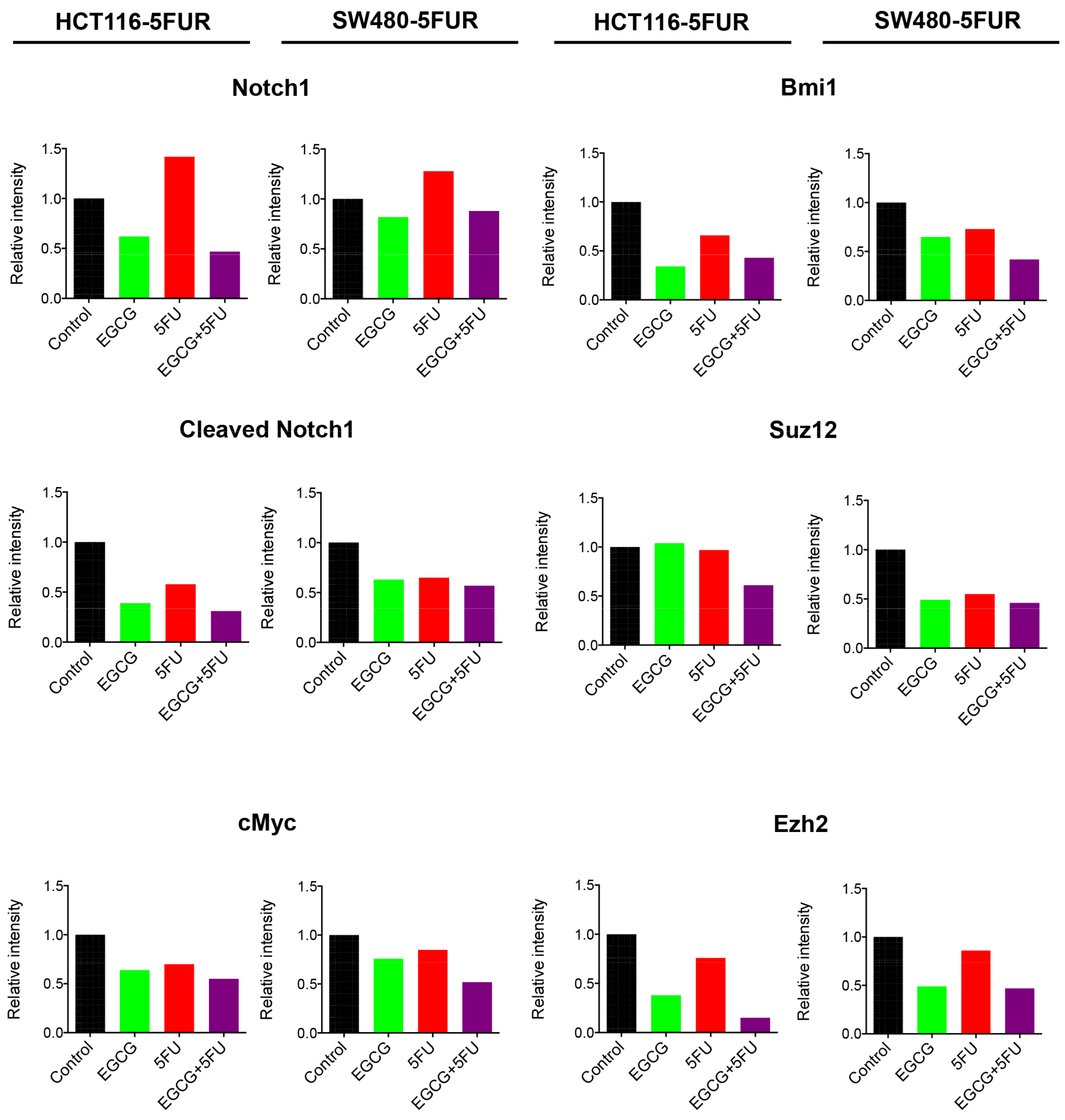


**Supplementary Figure S1: Quantitiave densitometry of the protein expression of Notch1, Cleaved Notch1, cMyc, Bmi1, Suz12 and Ezh2 in HCT116 and SW480-5FUR cell lines treated with EGCG and/or 5FU.** All measurements were represented as ratio between target protein/β-actin and control is normalized to 1.

**Supplementary Table S1: Primer sequences for gene expression assays**

| Primers | Sequences |
|---|---|
| Oct4 (F) | ACATCAAAGCTCTGCAGAAAGAACT |
| Oct4 (R) | CTGAATACCTTCCCAAATAGAACCC |
| Nanog (F) | CCGAAGAATAGCAATGGTGTGACG |
| Nanog (R) | AGGAGAATTTGGCTGGAACTGC |
| GAPDH (F) | ACCCAGACTGTGGATGG |
| GAPDH (R) | CAGTGAGCTTCCCGTTCAG |

**Supplementary Table S2: Antibodies used and their manufacturer's**

| Manufacturer | Antibody |
|---|---|
| Ezh2 | Cell Signaling |
| Bmi1 | Cell Signaling |
| Suz12 | Cell Signaling |
| CD44 | Abcam |
| Notch-1 | Cell Signaling |
| Cleaved Notch-1 | Cell Signaling |
| cMyc | BD Pharmingen |
| β-actin | Sigma |
